# *In vitro* and *in vivo* antiproliferative activity of metformin on stem-like cells isolated from spontaneous canine mammary carcinomas: translational implications for human tumors

**DOI:** 10.1186/s12885-015-1235-8

**Published:** 2015-04-07

**Authors:** Federica Barbieri, Stefano Thellung, Alessandra Ratto, Elisa Carra, Valeria Marini, Carmen Fucile, Adriana Bajetto, Alessandra Pattarozzi, Roberto Würth, Monica Gatti, Chiara Campanella, Guendalina Vito, Francesca Mattioli, Aldo Pagano, Antonio Daga, Angelo Ferrari, Tullio Florio

**Affiliations:** 1 Dipartimento di Medicina Interna, Sezione di Farmacologia, University of Genova, Genoa, Italy; 2 Centro di Eccellenza per la Ricerca Biomedica (CEBR), University of Genova, Genoa, Italy; 3 Istituto Zooprofilattico Sperimentale del Piemonte, Liguria e Valle D’Aosta, and National Reference Center of Veterinary and Comparative Oncology (CEROVEC), Genoa, Italy; 4 Dipartimento di Medicina Sperimentale, University of Genova, Genoa, Italy; 5 IRCCS AOU San Martino - IST, Genoa, Italy

**Keywords:** Breast cancer, Cancer stem cells, Metformin, Comparative oncology

## Abstract

**Background:**

Cancer stem cells (CSCs) are considered the cell subpopulation responsible for breast cancer (BC) initiation, growth, and relapse. CSCs are identified as self-renewing and tumor-initiating cells, conferring resistance to chemo- and radio-therapy to several neoplasias. Nowadays, th (about 10mM)e pharmacological targeting of CSCs is considered an ineludible therapeutic goal. The antidiabetic drug metformin was reported to suppress *in vitro* and *in vivo* CSC survival in different tumors and, in particular, in BC preclinical models. However, few studies are available on primary CSC cultures derived from human postsurgical BC samples, likely because of the limited amount of tissue available after surgery. In this context, comparative oncology is acquiring a relevant role in cancer research, allowing the analysis of larger samples from spontaneous pet tumors that represent optimal models for human cancer.

**Methods:**

Isolation of primary canine mammary carcinoma (CMC) cells and enrichment in stem-like cell was carried out from fresh tumor specimens by culturing cells in stem-permissive conditions. Phenotypic and functional characterization of CMC-derived stem cells was performed *in vitro,* by assessment of self-renewal, long-lasting proliferation, marker expression, and drug sensitivity, and *in vivo,* by tumorigenicity experiments. Corresponding cultures of differentiated CMC cells were used as internal reference. Metformin efficacy on CMC stem cell viability was analyzed both *in vitro* and *in vivo*.

**Results:**

We identified a subpopulation of CMC cells showing human breast CSC features, including expression of specific markers (i.e. CD44, CXCR4), growth as mammospheres, and tumor-initiation in mice. These cells show resistance to doxorubicin but were highly sensitive to metformin *in vitro.* Finally, *in vivo* metformin administration significantly impaired CMC growth in NOD-SCID mice, associated with a significant depletion of CSCs.

**Conclusions:**

Similarly to the human counterpart, CMCs contain stem-like subpopulations representing, in a comparative oncology context, a valuable translational model for human BC, and, in particular, to predict the efficacy of antitumor drugs. Moreover, metformin represents a potential CSC-selective drug for BC, as effective (neo-)adjuvant therapy to eradicate CSC in mammary carcinomas of humans and animals.

**Electronic supplementary material:**

The online version of this article (doi:10.1186/s12885-015-1235-8) contains supplementary material, which is available to authorized users.

## Background

Breast cancer (BC) is the most common and fatal malignancy in women [1]. Accumulating evidence supports the presence, within BC, of a subpopulation of tumor cells, named cancer stem cells (CSCs). These cells exhibit stem-like features, such as self-renewal, differentiation capacity, and are believed to represent the subpopulation responsible for the tumor-initiating activity and the resistance to antineoplastic agents [2,3]. *In vivo,* CSCs sustain tumor growth, reproducing the heterogeneity of the original tumor from which they are derived [4]. According to the current carcinogenesis theory, BC development and recurrence is driven by CSCs [5], and these cells represent the main pharmacological target for tumor eradication. Breast CSCs were initially characterized from surgically removed human tumors, although their isolation was possible only in a small percentage of postsurgical specimens [6]. However, since this first seminal study, most of the research on breast CSCs was carried out in established cancer cell lines [7,8], which were reported to contain putative CSC subpopulations. Conversely, only few studies were performed using cells isolated from tumor samples [9,10]. This limitation was likely a consequence of the CSC rarity within the tumor mass and the usually extremely small post-surgical specimens available for *in vitro* studies. A possible pitfall using cells expressing CSC signatures but isolated from continuous BC cell lines, is that they might include subsets of cells adapted to prolonged *in vitro* culture in the presence of high serum concentration that, overtaking the majority of the tumorigenic subpopulations, inadequately represent cancer cell heterogeneity. Moreover, due to genotypic and phenotypic alterations, these cells often show different drug responsivity from tumors *in vivo* [3,11].

The human BC cell subpopulation identified as CSCs is characterized by CD44^+^/CD24^low/−^ phenotype, the ability to grow *in vitro* as mammospheres maintaining a constant percentage of stem cells, high tumorigenicity *in vivo* [6,9], developing serially transplantable tumors in immunodeficient mice [12], indicative of long-term self-renewal ability [13,14]. Moreover, several BC CSC features are also relevant to metastasis, such as high motility, invasiveness, and resistance to apoptosis and drug treatments.

Recently, comparative oncology emerged as a relevant tool for pharmacological development in human cancer research. Spontaneous pet tumors represent important pre-clinical models of human cancers retaining the heterogeneous nature of tumors and allowing the validation of treatment strategies that will result beneficial to both human and animal patients [15,16]. These tumors, which develop in immunocompetent animals, at odd with those experimentally induced in laboratory rodents, display genetic, histopathological and biological features similar to the human counterpart, as well as the metastatic pattern and the response to therapy [17]. For example, spontaneous canine mammary carcinomas (CMCs) retain inter- and intra-tumor heterogeneity, as human cancer [18-20] but, due to the shorter life-span of dogs, they allow the evaluation of the natural course of the tumor and its pharmacological modulation after a shorter lag time than that required in human clinical trials. Thus, CMC is considered a reliable comparative model for human BC [21]. CMC is the most common neoplasm of female dogs, representing 50-70% of all tumors [22], and multiple deregulated genes and signaling pathways (PI3K/AKT, KRAS, PTEN, Wnt-beta catenin, MAPK, etc.) identified as responsible for its development, nicely resemble those observed in humans [19]. For example, the expression level of epidermal growth factor receptor (EGFR) in CMCs affects clinical prognosis [23]; HER-2 overexpression, occurring in about 20% of CMCs as in BC [24], or the loss of estrogen (ER) and progesterone (PR) receptors [25] are related to tumor progression. Moreover, triple-negative CMCs (lacking ER, PR and HER-2) show clinical-pathological characteristics associated with unfavorable prognosis, similarly to the triple-negative phenotype in women [26].

Because of the limited source of primary human BC tissues due to early diagnosis and multiple histopathological analysis required during and after surgery, and the lack of *in vivo* preclinical models that accurately reflect patients’ tumor biology, the study of pet spontaneous tumors may represent an innovative approach. However, this model is still underused and, in particular, studies on the role of CSCs in tumor development and treatment are lacking.

In veterinary research, putative CSCs have been identified in canine osteosarcoma, glioblastoma, acute myeloid leukemia, hepatocellular carcinoma [27-31], as well as in feline mammary carcinomas [32]. CSC-like subpopulations were isolated and partially characterized from canine mammary cancer continuous cell lines [33-35], mainly relying on *in vitro* observations, such as spheroid formation, cell surface antigens and aldehyde dehydrogenase (ALDH) activity, whereas isolation of CSCs from spontaneous canine mammary tumors have been described only in few studies [36]. Immunodetection of cells with CD44^+^/CD24^−^ phenotype in canine mammary tumor tissues, similarly to human BC CSCs, has been also reported [37], and CD44 expression has been associated with proliferation of cultured canine cancer cells [38]. Moreover, canine CSCs, isolated from the REM134 cell line, are resistant to common chemotherapeutic drugs and radiation, exhibiting epithelial-mesenchymal transition (EMT) phenotype [34].

Metformin is the first-line hypoglycemizing agent used for the treatment of type 2 diabetes (T2D) due to its efficacy and safety profile [39]. Epidemiological studies reported that metformin-treated T2D patients show reduced cancer incidence and mortality; furthermore metformin therapy seems to improve the clinical outcome of diabetic patients with cancer and to exert a protective anticancer effect in non-diabetic patients [40,41]. Thus metformin’s antitumor properties are currently tested in several clinical trials, mainly focusing on BC [42,43]. Preclinical *in vivo* studies reported that metformin reduces growth of BC xenografts in mice [44,45], and directly inhibits the proliferation of several BC [46,47] and other tumor [48] continuous cell lines, mainly interfering with CSC proliferation. However, in all these studies the effects of metformin, alone or in combination with doxorubicin or trastuzumab, were mainly evaluated in CSC-like derived from established lines [49-51].

Thus, the evidence of metformin activity in human BC CSCs is still limited, and a comparative approach studying CSCs from spontaneous dog tumors presents several advantages, including the retention of intra-tumor cell heterogeneity, an extremely relevant issue to identify pharmacological approaches with higher predictive validity when translated from preclinical to clinical setting. Moreover, since these tumors are often not treated before surgery, comparative oncology provides the unique opportunity in a preclinical model to map the nascent BC biology, without modifications induced by therapy pressure. Since CSCs are generally highly resistant to chemotherapy, drugs that successfully target this subpopulation may represent an effective therapeutic approach, and the analysis of efficacy on CMC may pave the way to the identification of clinically useful compounds in humans.

The aim of this study was to establish cell cultures enriched in CSCs from spontaneous CMCs, in order to provide a cellular model that may better reflect BC heterogeneity, pathogenesis and drug responses. Moreover, we tested the effects of metformin on CSCs isolated and characterized from spontaneous CMCs, providing evidence that these cells are highly responsive to *in vitro* and *in vivo* metformin treatment.

## Methods

### Canine mammary carcinoma tissues

Sixteen CMC samples were collected after surgical resection from the local network of free-lance veterinary practitioners (Genova, Italy), as described [32]. All histopathological diagnoses were reviewed and assessed according to the WHO International Histological Classification of Mammary Tumors of the Dog and Cat [52], and tumor grade was assigned [53].

### Immunohistochemistry

Immunohistochemistry (IHC) was as described previously [54]. Antibodies used were as follows: anti-EGFR (rabbit polyclonal; Cell Signaling Technology), anti-ER-α clone 1D5, anti-CD44, clone DF1485 and anti-Ki-67, clone MIB-1, (mouse monoclonal, Dako, Glostrup, Denmark) and anti-CD24 (goat polyclonal; SantaCruz Biothechnology). All these antibodies are directed against human epitopes but cross-react with the canine counterpart, as described [55-57]. Briefly, paraffin sections were deparaffinized and rehydrated, antigen unmasking was performed using citrate-antigen retrieval and Real Envision Detection System Peroxidase/DAB+, mouse/rabbit (Dako) was used for the detection according to the manufacturer's instruction. Counterstaining with haematoxylin concluded the processing. Images were captured using a Nikon Coolscope microscope. For CD44, ER-α and EGFR expression, both the intensity of immunoreaction and the percentage of positive cells were evaluated, and a score ranging from 0 to 3 was assigned (0 = negative, 1 = low positivity, 2 = positivity, 3 = high positivity). Ki-67 labelling index (Ki-67 LI) was evaluated, using anti-Ki-67 antibody, as the percentage of positive cell out of at least 1,000 neoplastic cells (Ki-67 LI: 1 = <10%, 2 = 10-50%, 3= > 50%), in 10 randomly selected microscopic fields. For each staining, a positive control was included (human breast cancer tissues), as well as a negative control, without the primary antibody or with rabbit/mouse IgG. Mitotic index (MI), as an indirect measure of cell proliferation, was evaluated as the number of mitotic figures per 10 high-power fields (HPF). Mitotic figures were counted in areas selected on the basis of the presence of good cellularity and high density of mitotic figures. Counting and semi-quantitative estimate of percentage of positive cells of IHC was evaluated and independently scored by two pathologists (A.R. and C.C.).

### Cell cultures

After surgery, tumor tissues were immediately processed for isolation of CSCs [32]. Tumor were finely minced and incubated in trypsin/collagenase for 20 min with agitation at 37°C, vigorously pipetted and cells passed through a 70 μm strainer (BD Biosciences, Milano, Italy) to obtain individual cells then plated in DMEM/Ham's F12 (1:1) medium, penicillin/streptomycin (100 U/ml), and glutamine 2 mM supplemented with 10% fetal bovine serum (FBS) (all from Lonza, Milano, Italy) or in a stem-cell permissive medium: (DMEM/Ham's F12 (1:1) without FBS, additioned with EGF (20 ng/ml), bFGF (10 ng/ml) both from Milteny Biotec (Bologna, Italy), 0.4% BSA (w/v, Sigma-Aldrich, Milano, Italy), and insulin (5 μg/ml, Sigma-Aldrich) to ensure stemness maintenance [9,32]. To induce differentiation, sphere colonies grown in stem-permissive medium were collected, dissociated into single cells, and shifted to complete FBS-containing medium (without growth factors) and cultured for at least 2 weeks [58].

### Cell immunophenotyping by immunofluorescence

To characterize CMC cells and visualize the expression of specific markers, immunocytofluorescence (IF) was performed [32]. Briefly, stem (mammospheres) and differentiated cells grown on coverslips were fixed in 4% paraformaldehyde, blocked in normal goat serum (Sigma-Aldrich) and the following antibodies were applied for 1 h at r.t.: CD44 (Cell Signaling Technology, Danvers, MA, USA), epidermal growth factor receptor (EGFR, Cell Signaling Technology), ER-α and pan-cytokeratin (pan-CK) (Dako). Secondary fluorescent antibodies, Alexa 488- and Alexa 568-conjugated goat rabbit/mouse-specific (Molecular Probes, Life Technologies, Monza, Italy), were added for 1 h at r.t. Nuclei were counterstained with 4',6-diamidino-2-phenylindole (DAPI, Sigma-Aldrich). Negative controls were included in the experiments by omitting primary antibodies. Images were captured by confocal laser scanning microscope (Bio-Rad MRC 1024 ES).

### MTT Assay

Cytotoxic effects were determined using the MTT [3-(4,5-dimethylthizol-2-yl)-2,5-diphenyltetrazolium bromide] (Sigma-Aldrich) reduction assay [59]. Briefly, viable cells (3x10^5^) were plated into 48-well plates and incubated overnight prior to exposure to increasing concentrations of metformin (0.1-100 mM) and DOX (0.01-5 μM) in the presence or absence of verapamil (10 μM). Cells were incubated with MTT solution 0.25 mg/ml for 2 h at 37°C, medium was removed and stain was solubilized in DMSO; absorbance was measured spectrophotometrically at 570 nm. Dose–response curves were generated and IC_50_ values were calculated using nonlinear regression curve fit analysis by Graph Pad Prism 5.2 (GraphPad Software, San Diego CA, USA).

### Clonogenic assay

Stemness of CMC CSCs was tested measuring the colony-forming ability of individual cultures [60]. Cells were seeded in 96-well plates, at <1 alive cell/well concentration; medium was changed twice a week. Plating accuracy was monitored under light transmitted microscope to confirm the presence of a single cell/well and exclude wells with dead or multiple cells. The number of wells that contained colonies was scored weekly up to 4 weeks.

### Doxorubicin uptake and intracellular distribution assay

The natural fluorescence of doxorubicin (DOX, Sigma-Aldrich) allows it to be localized by fluorescence microscopy *in vitro* [61]. CMC cells were seeded in 35 mm glass bottom dishes and allowed to growth o.n., then cells were exposed to 1 μM DOX for 20 h, in the presence or absence of 10 μM verapamil (Sigma-Aldrich). Cells were washed to remove non-associated drug and counterstained with the lipophilic membrane stain Vybrant DiO cell-labeling solution (Molecular Probes, Life Technologies, Monza, Italy). Images were captured by a DM2500 microscope equipped with a DFC350FX digital camera (Leica Microsystems, Wetzlar, Germany).

### *In vivo* xenograft studies

Female non-obese diabetic severe combined immunodeficient (NOD-SCID) mice (6–8 weeks old; Charles River, Calco MI, Italy) were used to evaluate tumorigenicity of CMC cultures [32,60]. Animals, housed in pathogen-free conditions, were handled in agreement with Italian regulations for the protection of animals used for scientific purposes and guidelines of the Ethical Committee for Animal Experimentation of the IRCCS-AOU San Martino-IST (Genova, Italy). Viable cells (4x10^5^) were collected by centrifugation, resuspended in matrigel (BD Biosciences) and pseudo-orthotopically injected in the subcutaneous fat pad. Mice were inspected weekly for tumor appearance by visual observation and palpation, thereafter were monitored for any discomfort and weighed until sacrifice. Tumor tissues were collected and dissociated to single cells as described above and after spheroid formation, cells were re-injected into new recipient mice to verify tumor-forming ability and incidence. The remaining tumor, fixed in 10% buffered formalin and embedded in paraffin was used for hematoxylin and eosin (H&E) histological evaluation. Treatments were started 7 days after inoculation of cells, in mice randomly allocated to groups receiving metformin or vehicle and continued for the next 6 months until the mice were killed. Metformin hydrochloride (Sigma-Aldrich) was dissolved in drinking water to attain the dosage of 360 mg/kg/die (see dose justification in Discussion). The water was changed every other day and measured for water intake. No toxicity signs were observed in the treated animals. Excised tumors were weighed, and portions were cultured or fixed for further studies.

### Plasma metformin measurement by high-performance liquid chromatography (HPLC)

Plasma concentrations of metformin in treated mice were determined by validated HPLC assay [62]. Mice blood samples (~0.3 ml) were taken under anesthesia by retro-orbital sinus bleeding, collected into heparinized tubes and centrifuged at 3000 x *g*. Metformin hydrochloride was used for calibration standards and as reference substance. Mouse plasma and standards were extracted with acetonitrile (0.5 ml) and reconstituted with water. An aliquot of each extracted sample (50 μl) was injected onto a Kontron HPLC system (Kontron Instruments, Munich, Germany), connected to an oven L-7350 column (Merck Darmstadt, Germany) and eluted with a mobile phase consisting of 20 mM K_2_HPO_4_ and acetonitrile (97:3 v/v) at a flow rate of 1 ml/min at 18°C. The UV detector (Kontron Instruments) was set at 236 nm (ABS 0.1, RT 0.1); the run lasted for 10 min. Results analysis was performed using the signal integration software KromaSystem 2000 (BIO-TEK Instruments Milano, Italy). The calibration curves of peak areas *vs.* concentrations of metformin were linear giving a correlation coefficient r^2^ = 0.999.

### Statistical analysis

All quantitative data were collected from experiments performed in triplicate, and expressed as mean ± s.e.m. Statistical analyses were performed using *t*-test (unpaired, two-tailed) or one-way ANOVA with Dunnett’s or Tukey’s post-tests, using GraphPad Prism 5.2 (GraphPad software). Differences were considered significant for p < 0.05.

## Results

### Clinical and histopathological characterization of CMCs

**Figure 1:**
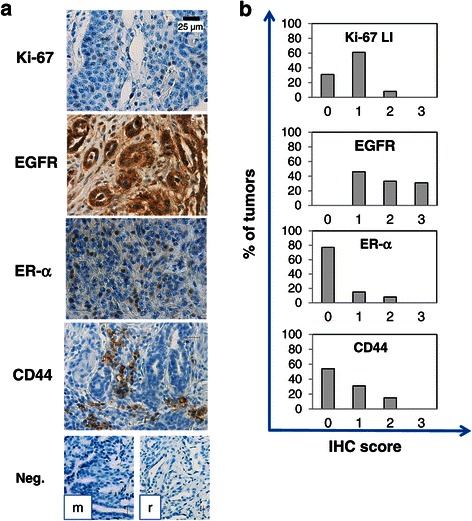
**Immunohistochemical expression of Ki-67, EGFR, ER-α and CD44 in canine mammary carcinomas. a)** Representative staining of a simple tubulopapillary carcinoma, analyzed for the expression of Ki-67 (nuclear staining), EGFR (both nuclear and cytoplasmic immunopositivity), ER-α (distinct nuclear staining) and CD44 (cytoplasmic staining). Antibody localization was done using HRP, with dark brown staining indicating the presence of the specific antigen. Original magnification 40X. Lower panels: negative controls (Neg.) obtained by using mouse (m) or rabbit (r) IgG as primary antibody. **b)** Distribution of Ki-67 labelling index (LI; 1 = <10%, 2 = 10-50%, 3= > 50% of positive cells/total tumor cells) and IHC scores (0 = negative; 1 = weak positivity; 2 = moderate positivity; 3 = strong positivity) for EGFR, ER-α and CD44 among CMC tissues.

Sixteen mammary carcinomas from female dogs were analyzed. Animal and tumor characteristics are reported in Table 1. Fresh tissue samples were divided into two parts: one was immediately fixed in 10% formalin and embedded in paraffin for histological diagnosis, and the other was dissociated to obtain primary cultures. Histological diagnoses (Table 1) included 7 simple, 7 complex and 2 anaplastic carcinomas. Tumor grade, highly predictive of epithelial tumors outcome in both dogs and in humans, was assessed by the presence or absence of tubule formation, nuclear pleomorphism, and the number of mitosis per 10 HPF, and revealed 5 grade II and 11 grade III tumors. Notably, histopathological features of this series of CMCs covered a wide range of representative canine tumor subtypes and pathological/prognostic signatures that overlap most common human BC profiles. IHC was performed in all cases to evaluate the expression levels of the proliferation marker Ki-67, and to identify CD44-expressing cells, as potential CSCs. To further characterize tumor phenotype relevant receptors involved in mammary tumorigenesis, such as ER-α and EGFR, were analysed. Representative IHC images are shown in Figure 1a. Tumor immunoreactivity for these markers was assessed by semiquantitative IHC score, as described in Materials & Methods (Figure 1 b). Ki-67 expression was classified as “low” in ~60% of tumors, while about 10% were categorized as “intermediate” (range 10-50% of positive cells). The analysis of the expression pattern of EGFR showed a marked immunopositivity, although detectable at different levels (46% score 1, and 33% score 2 or 3) in all tumors, while ER-α was detected in a limited number of samples (about 25%). Interestingly, in agreement with previous studies [63], CMCs showed different ER-α localization, being either clearly nuclear or mainly localized in the cytosol (Additional file 1: Figure S1).

**Table 1:** **Clinical data and histopathology of canine mammary carcinomas**

**Total n.**	16		
**Mean Age**	10 yrs. (range 6–14)		
		**N.**	**%**
**Breed**	Pure	9	56
	Mixed	7	44
**Sex**	F	14	88
	FS	2	12
**Histology**	CT	2	12
	CTP	3	20
	CSP	2	12
	CC	7	44
	CA	2	12
**Grade**	II	5	32
	III	11	68
**Localization**	M2	1	6
	M3	1	6
	M4	1	6
	M5	8	50
	n.a.	5	32

Histology: CT, carcinoma-simple, tubular; CTP, carcinoma-simple, tubulopapillary; CSP, carcinoma-simple, papillary; CC, carcinoma complex; CA, carcinoma, anaplastic.

Sex: F: female, unspayed; FS: female, spayed.

Localization: M2, caudal thoracic; M3, cranial abdominal; M4, caudal abdominal; M5, inguinal.

n.a.: not available.

CD44 expression, a potential CSC marker, as determined in human BC CSC (phenotype CD44^+^/CD24^-/low^), was detected in scattered cells (score 1) in the 31% of the cases, or in limited tumor regions (score 2) in 15%, while 54% of tumors were negative. However, as previously reported [32], CD44 is likely underestimated by random IHC sampling, due to the non-homogeneous expression within tissues, the predicted rarity of these cells, their potential localization in stem cell niches and the lack of analysis of serial sections of the samples. The expression of CD24 was checked in our series of CMC tissues but no stained cells were detected, accordingly to previous observations [32,37].

### Isolation and *in vitro* expansion of cancer stem-like cells from CMC specimens

**Figure 2:**
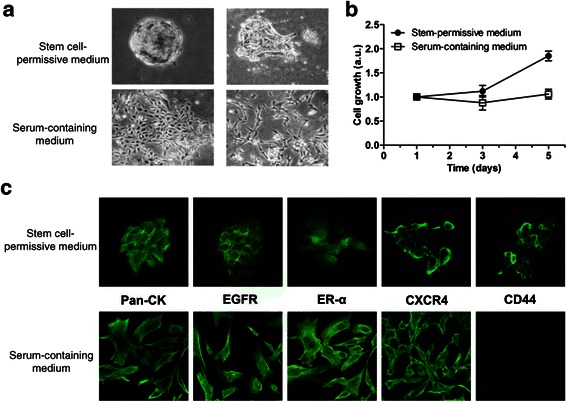
**Morphological appearance, proliferation and phenotyiping of CMC cultures. a)** Morphological changes of floating mammospheres/clusters in stem cell-permissive medium (upper panels) from a cobblestone-like morphology to spindle-like cells in adherent monolayers (lower panels), after differentiation for 15 days in serum-containing medium. Phase-contrast images, original magnification 10X. **b)** Representative growth curves of CMC cells selected under stem cell-permissive or differentiation medium, showing the in *vitro* proliferative potential of cultures. Arbitrary units (a.u.) are referred to the number of living cells at day 1. Data represent the mean ± s.e.m. **c)** Enrichment in CD44^+^ cells and marker expression profile of CMC cells grown in stem cell-permissive or differentiation medium. Immunofluorescence analysis of pan-cytokeratin (Pan-CK), EGFR, ER-α, CXCR4 and CD44 in CMC spheroids (upper panels) and after 15-day exposure to serum-containing medium. Images from confocal microscopy, original magnification 100X.

Primary CMC cultures were obtained from fresh tumors by mechanical disaggregation and enzymatic digestion. Single-cell suspensions, obtained by cell strainer filtration, were plated in stem-permissive culture medium (serum-free and supplemented with bFGF and EGF). In these conditions, cells grow as low/non-adherent spherical clusters of cells (mammospheres). Morphologically, CMC mammospheres, formed by cobblestone-like epithelial cells, exhibited features of both floating spheroids of variable sizes and partially attached irregular aggregates (Figure 2a, upper panels). In parallel, aliquots of primary culture from the same tumors, were grown in DMEM-F12 supplemented with 10% FBS, without growth factors. These culture conditions do not allow the selection for CSC-like, favoring the proliferation of non-tumorigenic differentiated mammary carcinoma cells. In these conditions, cells grow *in vitro* as adherent monolayers, showing a predominance of spindle-like morphology, but are not able to generate mammospheres (data not shown). CSC-like differentiation ability was tested by shifting mammosphere-derived cells in serum-containing medium (10% FBS, devoid of growth factors) for at least 2 weeks. In these conditions, cells from disaggregated spheroids adhered to the substrate and acquired a spindle-like morphology, resembling primary CMC cultures originally grown in FBS-containing medium, immediately after isolation (Figure 2a, lower panels). To assess the pattern of proliferation *in vitro* of CMC cells from both culture conditions, growth-curves were generated according to the absorbance values, in MTT assays. Proliferative activity of cells in stem-permissive medium was markedly enhanced when compared with differentiated cells, whose growth rate reached a plateau stage early after seeding (Figure 2b).

### Phenotypic characteristics of canine mammary carcinoma cells cultured in stem-permissive medium

To verify whether primary CMC cultures grown in stem cell-permissive medium are indeed enriched in CSCs, we characterized the phenotype of these cells by IF (Figure 2c, upper panels). All stem-like cultures exhibited pan-cytokeratin expression, consistent with their epithelial origin. As self-renewal of human and animal mammary cancer stem cells involves a diverse network of regulatory mechanisms, including the signaling pathways of EGFR, CXCL12/CXCR4 and ER-alpha, we analysed whether these proteins are expressed in CMC cultures.

We observed a marked positivity for EGFR within spheres and single cells growing under stem-permissive/serum-free conditions indicating that EGFR immunopositivity observed in tissue sections was retained after CSC enrichment *in vitro*. CXCR4 was also expressed in CSC cultures showing predominant membrane localization. Conversely, the expression of ER-α was not detected in all the cells of the analyzed spheroids, and 30% of them were completely negative, in agreement with IHC analysis (see Figure 1). Importantly, the expression of CD44, previously reported as signature of human breast CSCs [6], was detected in all cultures, providing evidence for enrichment in stem-like cells and validating their identification in corresponding CMC tissues (Figure 2c). Differentiated cells (shifted to serum-containing medium) conserved similar expression profiles of all the markers, with the remarkable exception of CD44, which was undetectable after differentiation (Figure 2c).

### CMC stem cells are tumorigenic *in vivo*

**Figure 3:**
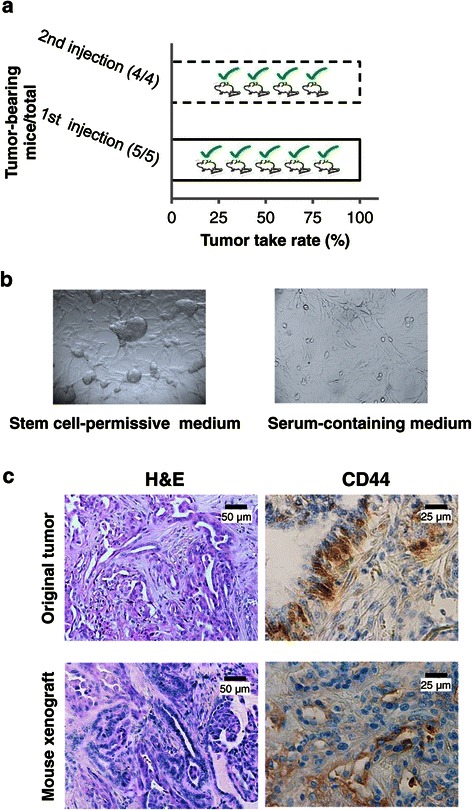
**Tumorigenicity of stem-like cells derived from CMC cultures. a)** Tumor-forming rates of mammosphere-derived cells from CMCs. Cells were pseudo-orthotopically injected into NOD/SCID mice, and tumor development was monitored. Tumor take rate was 100% after the 1^st^ injection. Tumorigenicity rate was steadily up to 100% when cells recovered from primary xenografts were cultured in stem-permissive conditions and re-injected into mice fat pads. **b)** Cells derived from mice xenografts appearance after *in vitro* culture. As the original cultures derived from canine carcinomas, cells grow as partially attached or floating spheroids in stem conditions, while upon differentiation are able to attach to the substrate and grow as monolayer. Phase-contrast images, original magnification 20X. **c)** Canine mammary stem-like cells fully recapitulate the tumor of origin when transplanted into immunodeficient mouse. Histopathologic examination of implanted tumors: representative H&E staining reveals the typical appearance of tubulopapillary carcinomas both in the original canine tumor and the corresponding mice xenograft; immunohistochemical staining for CD44 shows the presence of rare, but observed in each tumor tissue, positive cancer cells (original magnification 40X).

At present, the gold standard assay to assess CSC potential is the transplantation of prospectively identified cancer cell subpopulations into immunodeficient mice to assess tumorigenicity, phenocopying the original tumor [64]. Mammosphere-derived CMC stem-like cells (4x10^5^ cells/mouse), isolated from a grade III tubular carcinoma, were pseudo-orthotopically injected in NOD/SCID mice. Tumor development was daily monitored, and animals were sacrificed after 23 weeks, when symptoms of physical or behavioral deficits developed, due to tumor size. Transplanted CMC cells achieved high take rate (up to 100%) after first injections in mice (Figure 3a). Cells derived from tumor explants, cultured again in stem cell-permissive medium, grew as partially attached aggregates or floating spheroids, as described for cultures derived for CSC-like cells from the original tumor (Figure 3b), retaining the tumor-initiating ability after 2^nd^ injection into new recipient mice, efficiently generating secondary tumors (Figure 3a). However, when shifted in FBS-containing medium these cultures exhibited differentiated morphology and monolayer growth (Figure 3b). Histopathological analysis of original and mouse xenograft tumors revealed that CSC-like derived tumors closely reproduced the histotype of the original CMC (i.e. carcinoma predominantly organized in tubular structures) (Figure 3c, left panels), including CD44 expression in scattered tumor cells evaluated by IHC (Figure 3c, right panels). Similar results were obtained injecting CSCs from grade III tubulopapillary carcinoma or complex carcinoma (data not shown).

Collectively these data confirm the tumor-initiating capacity of isolated CMC CSCs, and their ability to recapitulate the phenotype of the original tumor *in vivo.*

### CMC stem cells are resistant to doxorubicin: reversal of the resistance by verapamil

Human BCs develop numerous mechanisms of resistance to chemotherapeutic drugs, allowing them to survive conventional therapies and to drive tumor recurrence and metastasis. CSCs are believed to represent the main source of drug-resistant cells [2].

**Figure 4:**
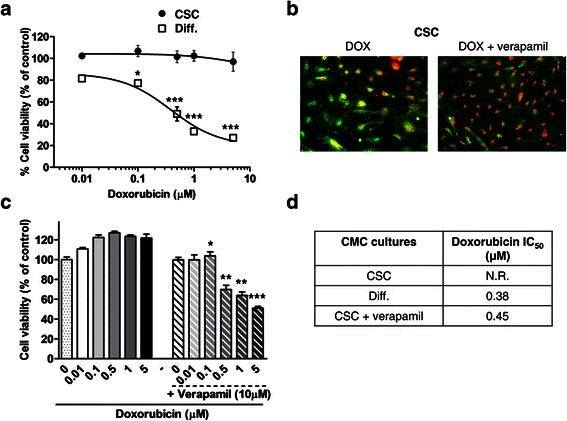
**CMC stem cells are resistant to doxorubicin: reversal by verapamil pretreatment. a)** Cumulative dose–response curves of the effects of doxorubicin (DOX) on cell viability, measured by MTT assays, in CSC (CMC CSC) and differentiated (CMC DIFF) canine mammary carcinoma (CMC) cells. A statistically significant reduction in cell viability of CMC DIFF was observed (*p < 0.05 for 0.1 μM DOX, ***p < 0.001 for higher concentrations *vs*. control), while CMC CSC were not affected. Data represent the mean ± s.e.m. **b)** DOX intracellular distribution in CMC CSC untreated (left) or treated (right) with the calcium channel blocker verapamil, to inhibit ABC transporter activity. CSCs were labelled with the lipid green dye DiO to highlight cell shape. Subcellular DOX fluorescence (red) localization is mainly confined to cytoplasm (co-localization with DiO, yellow) of resistant cells, while the fluorescent accumulation of DOX in the nuclei is markedly increased by verapamil. Original magnification 20X. **c)** Dose–response analysis of verapamil on the cytotoxic activity of DOX: reversal of resistance was significantly achieved starting from 0.1 μM (*p < 0.05, **p < 0.01; ***p < 0.001 *vs.* respective value of DOX alone). Data represent the mean ± s.e.m. **d)** Mean IC_50_ values calculated using nonlinear regression curve fit analysis in CMC cells exposed to DOX alone or in combination with verapamil.

To investigate whether these mechanisms are functional in CMC stem cells, we monitored cell viability in 3 different CMC CSC cultures exposed to doxorubicin (DOX, 0.01-5 μM), a standard drug for human BC. CSCs were resistant to DOX (after 48 h no reduction of cell viability was observed even at the highest concentration tested, Figure 4a). Conversely, differentiated cells showed high responsivity with maximal cell viability reduction (−80% *vs*. untreated cells) observed after 48 h, and a mean IC_50_ of 0.38 μM (Figure 4a and d). Comparison of dose–response curves obtained from differentiated and stem-like cells showed a highly significant statistical difference (p = 0.019, ANOVA).

One of the most common mechanism of resistance to DOX, representing also a key feature of CSC subpopulation, is the overexpression of ATP-binding cassette transporters (ABCB1, ABCG2, and ABCC1) whose activity leads to cell extrusion of cytotoxic drugs.

To gain further insights into the role of ABC pumps in drug resistance, we assessed the intracellular distribution of DOX autofluorescence in CMC CSCs. We expected that high activity of multidrug resistance transporters would change the pattern of DOX localization and, specifically, decrease its nuclear accumulation. As shown in Figure 4b, after 24 h of treatment, DOX either was extruded from the majority of the CSCs or, when entered cells, it was pumped out from the nucleus accumulating in perinuclear/cytoplasmic structures, as evidenced by the merge of vital cell dye DiO (green) and DOX (red) fluorescences, resulting in colocalization (yellow) (Figure 4b, left panel). Inhibition of ABCB1 pump function, by the calcium antagonist verapamil (10 μM), prevented DOX exclusion, resulting in its accumulation within the nucleus of all the cells (Figure 4b, right panel), a similar pattern to that observed in DOX-sensitive differentiated cells (data not shown). These results, suggesting ABCB1 involvement in CSC DOX resistance likely due to reduced access to nuclear targets, were confirmed by MTT experiments. CMC CSCs treated with DOX plus verapamil for 48 h acquired a significant responsivity to the cytotoxic drug (p = 0.025 *vs*. DOX alone, ANOVA) (Figure 4c), reaching a mean IC_50_ of 0.45 μM, a value that was almost superimposable to that observed in differentiated CMC cells (Figure 4d). Verapamil had no significant effects on the subcellular distribution or accumulation of DOX in differentiated cells that however showed a nuclear localization of the drug also in the absence of verapamil (data not shown).

### Metformin inhibits CMC stem-like cell viability *in vitro*

**Figure 5:**
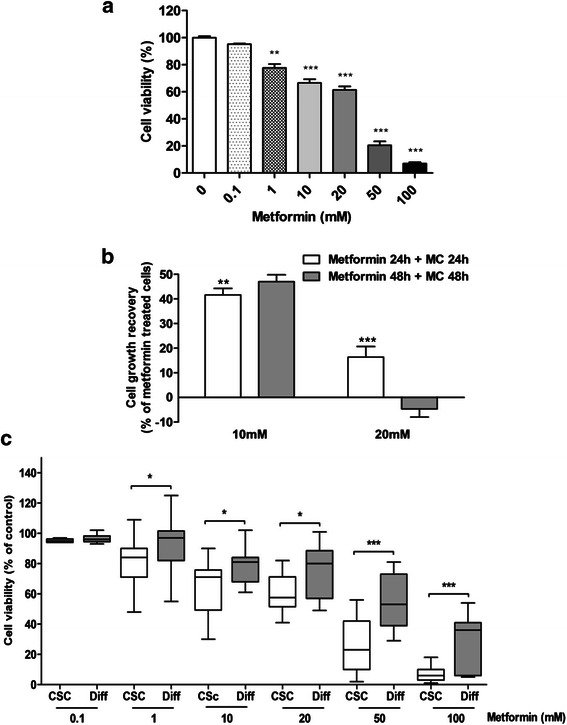
**Metformin inhibits CMC stem cell viability*in vitro.*a)** Dose–response of metformin on cell viability, measured by MTT assays, in CMC CSC cultures. A statistically significant reduction in cell viability was observed (**p < 0.01 for 1 mM metformin and ***p < 0.001 for higher concentrations *vs*. control). Data represent the mean ± s.e.m. **b)** Cell growth recovery assay in CSCs treated with metformin (10 and 20 mM) for 24-48 h and followed by drug withdrawal and growth in drug-free medium (medium change: MC) for further 24-48 h. Results from MTT assays show cytostatic and cytotoxic effects of 10 mM and 20 mM metformin, respectively (**p < 0.01, ***p < 0.001 *vs.* corresponding treated cultures). **c)** Box and whiskers plot of metformin effects on viability of CSC and differentiated (Diff) CMC cultures: boxes extend from the 25^th^ to the 75th percentile, lines indicate the median, whiskers extend to minimum and maximum data points. A significantly higher sensitivity of CSC cultures than corresponding differentiated cells is evident stating from 1 mM metformin for 48 h (*p < 0.05; ***p < 0.001 *vs*. corresponding differentiated cells).

While CSCs are resistant to most conventional cytotoxic drugs, recent data suggested their possible sensitivity to drugs such as metformin [65,66]. To delve deeper in this issue, we evaluated the effects of metformin in all the 16 CMC cultures. Metformin caused a significant reduction of cell viability (p < 0.001, ANOVA), in a dose-dependent manner, starting from the concentration of 1 mM, after 48 h of treatment, and reaching a mean IC_50_ of 12.59 ± 3.49 mM (range 0.40-31.22 mM) (Figure 5a). This effect was mainly cytostatic up to the concentration of 10 mM since, in cell growth recovery experiments, a significant proliferation was observed, after drug wash-out, in cells pretreated for 24 h and 48 h with metformin (p = 0.007 and p = 0.0004, respectively; Figure 5b). However, using higher concentrations (20 mM) growth recovery was minimal after 24 h of treatment, and completely abolished after 48 h, indicating a cytotoxic activity (Figure 5b).

Differentiated cell cultures were obtained in parallel with stem-like cells from 13/16 tumors. Differently from what reported in BC cell lines in which metformin was highly selective for the CSC component [49], metformin also affected viability of CMC cells grown in FBS-containing medium, at concentrations higher than 10 mM (Figure 5c), showing a higher mean IC_50_ value, but not statistically different from that obtained in the CSCs isolated from the same tumors (24.5 mM *vs.* 17.8 mM). However, comparing viability curves of both culture conditions, we found that CSCs display a significantly higher reduction of viability than differentiated cultures at all the tested concentrations, with the exception of the lowest one (0.1μM; Figure 5c), suggesting that metformin actually targets with higher efficacy CSC-like cells, while doxorubicin more efficiently blocks differentiated cell proliferation.

### Metformin impairs the growth of CMC stem-like cell *in vivo*

**Figure 6:**
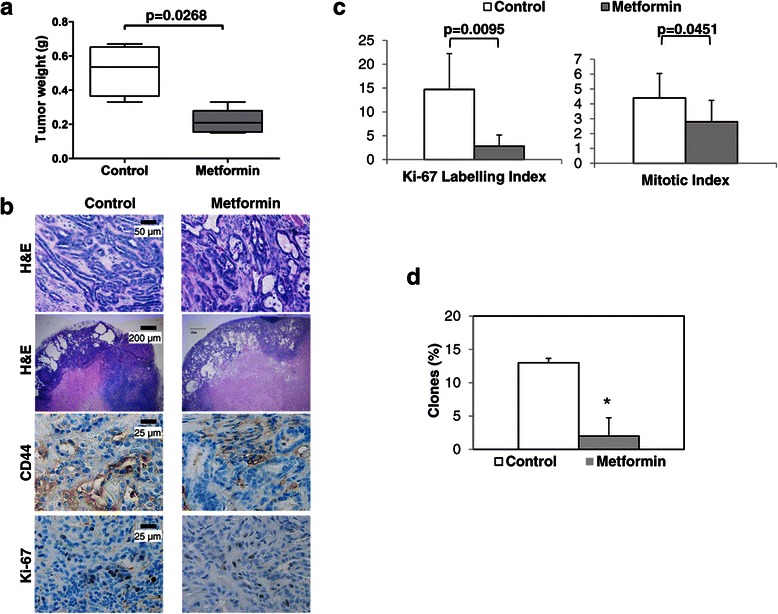
**Metformin inhibits canine mammary CSC proliferation*in vivo.*a)** Metformin significantly suppresses CMC CSC tumor growth. The box-plot whiskers extend from the lowest to the highest tumor weight value, from tumors explanted from control or metformin-treated mice. A significant statistical difference between the two groups is reported within the graph. **b)** Histopathological analysis of control and metformin-treated excised xenografts. Representative images of mouse-developed canine mammary tubular carcinoma analyzed by histopathological and immunohistochemical staining. H&E (upper panels, 20X) shows larger areas of necrotic tissue in treated tumors than in xenografts derived from untreated mice (4X). Cells expressing CD44 and Ki-67 are evidenced by immunohistochemistry in lower panels (40X). **c)** Metformin reduces the proliferative potential of mammary carcinoma cells within tumor: microscopic examination and quantification of Ki-67 and mitotic counts were significantly reduced by metformin. **d)** Metformin significantly decreased clonogenicity of xenograft-derived cells surviving long-term metformin exposure *in vivo* (*p<0.05).

In order to directly test the *in vivo* effects of metformin on CMC growth, metformin was orally administered in drinking water to 6 NOD-SCID mice xenografted with CSCs (4x10^5^) isolated from 1 tubular and 1 tubulopapillary carcinoma (three mice per histotype), while other 6 mice, injected with CSCs from the same tumors (3 each), were used as untreated controls. Tumors were allowed to grow till animals presented signs of physical distress, when the mice were sacrificed. None of the animals exhibited signs of drug-related toxicity. After 6 months, all mice were sacrificed, tumors explanted, weighed, and divided in two samples, one analyzed by IHC and the other dispersed to single cells cultured *in vitro*. Metformin plasma concentrations were measured in all treated mice, showing a mean level of 6.9 μg/ml (range of 4.5-13.32), corresponding to ~41 μM a value compatible with therapeutically efficacious concentration in humans. Metformin caused a significant reduction of tumor growth (−62% of tumor weight, p = 0.026 *vs.* untreated controls; Figure 6a). Xenografts morphologically resembled the tumor of origin, but H&E staining highlighted the presence of large necrotic areas in metformin-treated tumors that were absent or extremely small, in control tumors (Figure 6b). Importantly, a lower content of CD44-expressing cells was observed in metformin-treated xenografts than in untreated tumors (Figure 6b), although a precise quantification was not possible due to the heterogeneous presence of these cells within the tumor mass (see also comments to data reported in Figure 1). Conversely, the proliferative activity, assayed by quantification of Ki-67-LI, confirmed a highly statistically significant reduction in metformin-treated tumor sections as compared with controls (Figure 6c). Mitotic index, a measure of cell proliferation considered a strong predictor of the clinical outcome for several human and canine cancers, also revealed a significant decrease after metformin administration (Figure 6c).

Finally, to define the impact of *in vivo* metformin treatment on CSC viability, we analyzed the clonogenic activity of individual CMC cells in *ex vivo* experiments, as a CSC-based *in vitro* index of *in vivo* tumorigenicity. Cells, derived from treated and control tumors, grown in stem-permissive medium, were plated as single cells and allowed to give origin to clones for up to 30 days. About 13% of the cells derived from untreated tumors retained clonogenic activity, a percentage compatible with the CSC levels within the tumor mass, while only 2% of the cells from metformin-treated xenografts retained this stem-defining feature. These data clearly suggest that *in vivo* metformin treatment powerfully affect the survival of CSCs. In addition, we observed that CMC cultures derived from untreated and chronically metformin-treated xenografts, grown in stem cell-permissive conditions, were similarly sensitive to the antiproliferative activity of metformin (IC_50_, 22 and 26.6 mM, respectively) showing superimposable dose–response curves (p = 0.3513; data not shown), providing evidence that desensitization of the antiproliferative mechanisms activated by metformin does not arise during prolonged *in vivo* treatment.

## Discussion

The hierarchical model of carcinogenesis implies that only a small subset of tumor cells, named CSCs or tumor-initiating cells, drives tumor development and progression, determining drug responsiveness. Conversely, differentiated tumor cells, incapable of long-term self-renewal but representing the majority of the cells within hematologic and solid cancers, are not tumorigenic. Although most studies addressing this issue relied on established cancer cell lines rather than primary cultures, it is now clearly evident that putative CSC identification and usefulness is strictly coupled with powerful and systematic *in vitro* isolation and *in vivo* transplant of CSCs derived from fresh tumor tissues [64].

In the present study, we isolated and characterized CSCs from CMC surgical tissues, by selection of primary CMC cultures in stem cell permissive, serum-free medium for several passages *in vitro*. CMC-derived stem cell-like spheroids express CD44, as well as other BC related proteins, such as EGFR and CXCR4, and showed high resistance to DOX when compared to the corresponding CMC differentiated cells. Xenografts in NOD/SCID mice demonstrated that CMC CSCs derived from mammosphere disaggregation, successfully initiate tumor formation.

Breast cancer remains a major clinical challenge with high mortality both in humans and companion animals [1,25]. Advancement in understanding tumor biology and public awareness campaigns led, in most cases, to early BC diagnosis and neo-adjuvant treatments were developed in women. While the better clinical management significantly improved the overall prognosis of BC patients, only small fragments of fresh tissues, frequently already exposed to intensive cytotoxic therapy, are available from excised human tumors for experimental purposes, also because large amounts are required for intra- and post-surgery histopathological analyses. On these premises, a major strength of our study is the use of spontaneous dog tumors as a source of biological material necessary to isolate mammary CSCs. Indeed, to improve the knowledge of *in vitro* and *in vivo* biological features of CSCs and their drug responses, allowing the translation of preclinical findings into effective human clinical trials, cell models that faithfully mimic BC cell heterogeneity are an absolute requirement. Spontaneous tumors of companion animals, such as CMC, are rather frequent in the clinical veterinary practice [22] and thus may provide a unique opportunity as a model for human cancer translational research [15]. In contrast to experimental tumor models in mice, CMCs develop naturally, reproducing the same environmental and genetic aetiology as occurs in humans, grow in immunocompetent organisms [67] and share strong clinical (e.g. hormonal dependence, age of onset, histological appearance, prognostic factors, course of the disease) and molecular (e.g. tumor genetics, overexpression of steroid receptors, proliferation markers, EGF, p53 mutations, metalloproteinases, cyclooxygenases) similarities to human BC [20,22].

Additionally, human and canine mammary tumors show similar responses to conventional anticancer agents and, more importantly, both display inter/intra-patient tumor cell heterogeneity [68]. The enrichment in CSCs has been largely demonstrated in established human and canine mammary cancer cell lines [5]. Here, we reinforce the hypothesis of the stem cell basis for mammary tumors, achieving isolation of such a population from surgical samples. Features of CSCs include: (i) self-renewal capacity, (ii) stem marker expression, (iii) ability to reproduce the original tumor after xenografting, and (iv) chemo-resistance. Therefore, we verified these prerequisites in CSCs derived from CMCs. Cell surface marker CD44 [6], and the expression of the enzyme aldehyde dehydrogenase (ALDH), partially overlapping within this subpopulation [69], were assumed as criteria for CSC-enrichment and stem signatures in human BC, correlating to the tumor-initiating ability. However, it is currently debated whether these markers univocally identify CSCs in all BCs [70,71]. Breast CSC plasticity induced by tumor microenvironment, which allows these cells to undergo reversible EMT, may influence distinct marker profiles [72]. Nevertheless, CD44 was identified as major target using CD44 antibodies [73] or inducing CD44 down-regulation [74,75], to inhibit CSC proliferation and migration and overcome drug resistance.

In dogs, limited studies on mammary CSCs are available, thus their phenotyping is mainly derived from commonly employed human markers [33,36], reproducing similar controversies: spheres from primary tumors show the expression of CD49f (integrin α6) and CD29 [36] while spheres derived from canine cell lines express CD44 and CD133 and/or Sox2 and Oct4 [33]. Moreover, CD44 was associated to proliferative activity of cultured canine cancer cells [38,71]. CMC cultures, analysed after *in vitro* enrichment in stem-like cells, display CD44 expression in a high number of cells, while, in the tumor of origin, CD44^+^ cells are confined in randomly spotted tissue areas, as described for human samples [71], explaining, as previously discussed, the apparent high percentage of CD44 negative tumors. However, although this is a non-defining criterion for CSC identification [76] and we did not select CMC CSCs on the basis of markers’ expression, we observed a depletion/decrease of CD44^+^ cells upon CMC CSC differentiation (shifting cell cultures in serum-containing medium), confirming that this marker labels some CSC-like populations in culture. In addition, we analysed the expression of genes associated, in human and pet mammary cancers, with stem/progenitor cell survival, self-renewal and tumor aggressiveness, such as EGFR, ER-α and CXCR4 [54,77,78]. Similarly to the original tumors, CMC mammospheres quite homogenously express EGFR confirming its relevance in mammary cancer biology, and CXCR4, whose signalling regulates BC stem cell activities and metastatic potential [54,79]. Conversely, ER-α positivity was less commonly detected in isolated CMC stem-like cells and in tumor sections. The pattern of expression of these proteins did not change in the corresponding differentiated CMC cultures, showing that both the bulk of CMC cells and CSC-enriched cultures retain *in vitro* the same distinctive factors, as master regulators of tumor formation and growth. Thus, CSC cultures isolated from CMCs reproduce the tissue heterogeneity, covering different histopathological types and maintaining in culture the phenotype observed *in vivo*.

CSC biomarker expression [80] and the ability to growth in serum-free medium as non-adherent spheroids [81] are variably identified features in human and canine mammary tumors, reflecting intra- and inter-tumor heterogeneity but do not systematically define CSCs [82]. Thus, functional validation, by means of *in vivo* tumorigenicity experiments, is actually the more reliable tool to corroborate the definition of cell populations as CSCs (tumor-initiating cells). We demonstrate that CSCs isolated from CMC retain tumor-initiating activity, by serial transplantation in NOD-SCID mice (CSCs were xenografted, recovered and re-transplanted to form new tumors in new recipient mice) achieving about 100% of take rate after both 1^st^ and 2^nd^ injection, reforming the heterogeneous population of CMC cells within the xenografts. Another biological property generally ascribed to CSCs is drug resistance, as they more efficiently survive therapy than differentiated cancer cells. Indeed, the major challenge in BC treatment is the targeting of CSCs usually refractory to conventional drugs both in humans and in dogs [34]. This underlines the need of novel preclinical models to test drug effects on CSC biological features such as increased drug-efflux and DNA repair ability, and resistance to apoptosis, that contribute to drug resistance. In fact, using these mechanisms CSCs survive therapies contributing to recurrence and progression, after the initial remission caused by the differentiated tumor cell death [83]. Drug-resistance of CMC CSCs is therefore similar to that observed in human BC stem cells, whose content within the tumor mass is increased by chemotherapy [83], and in CSCs isolated from a CMC cell line [34]. Therefore, although cytotoxic treatments reduce the bulk of the tumor, they may not affect the most important target: the CSCs. In this study we developed a preclinical model reproducing this *in vivo* condition. CMC CSCs survived DOX treatment, at odd of differentiated cultures isolated from the same tumors, likely due to transporters that pump out the drug from the nuclei or outside the cells.

The need of drugs effectively targeting CSCs inspired new approaches for anti-cancer drug discovery and in particular the so-called “drug repositioning”. It was reported that the biguanide metformin specifically inhibits self-renewal and proliferation of CSC from several tumors [66,84,85], including BC [49,51,86,87]. Starting from early evidence using different biguanides [88-90], the antitumor potential of metformin is now well-established [65,91]. Epidemiological studies showed that diabetic cancer patients may benefit of metformin treatment and, on these bases, several clinical trials are ongoing [92]. Recently, the inhibition of chloride intracellular channel 1 (CLIC1) activity was identified as a specific molecular mechanism by which metformin affects only CSC viability, sparing normal stem cells [93], making this molecule particularly interesting as novel anticancer drug. We show that canine CSCs are not sensitive to DOX cytotoxicity, but highly responsive to metformin *in vitro*, while differentiated tumor cells are only partially affected by metformin, although highly responsive to DOX. These data confirm that combined treatment with metformin and conventional cytotoxic drugs may provide a therapeutic advantage. In fact, while metformin is clearly preferentially active on CSCs, a successful therapy will require targeting of both undifferentiated CSCs and differentiated non-CSCs [94], since the reverse transition of non-CSCs into CSC subpopulation was also reported [95]. Mean metformin IC_50_ values obtained in our experiments is about 10 mM, ranging from 0.40 to 31 mM in cells from different tumors, evidencing significant variability in metformin activity among individual dog-derived cells, as expected for the inter-patient tumor heterogeneity, although we remark that viability of CSCs from all 13 CMCs analyzed was impaired. These IC_50_ values are in line, and in some cases lower, with those of most of the previous studies reporting antitumor activity of metformin (range 1–30 mM) [44,87,96,97]. However, metformin concentrations used *in vitro*, exceeding those achieved *in vivo* in T2D patients (10–30 μM range) [39], are still a debating issue, due to the concern whether these *in vitro* data might be relevant for translation to clinics [98]. We acknowledge that the concentrations used are higher than metformin steady state plasma levels in T2D patients, however, the discrepancy between clinical and *in vitro* conditions could be less significant considering the following factors: (a) metformin concentrations in tissues are several-fold higher than those in blood because of tissue accumulation [99], thus actual intra-tumor concentrations should compare with *in vitro* results; (b) medium supplements, required to maintain tumor cell proliferation in culture (i.e. high concentrations of glutamine and glucose), reduce cell sensitivity to metformin [4,100,101]; (c) tumors often show increased cationic transporters compared their normal counterparts [91] further favoring tissue accumulation, (d) longer exposure of cell cultures to metformin (15–18 days) shift metformin-antitumor effect to a lower threshold [44]. In fact, it was reported that in human glioblastoma CSCs, short-term experiments (24-72 h, as here reported) show metformin anti-proliferative activity in the mM range [44,93,102], but increasing drug exposure to 15 days metformin efficacy was evident already at 10 μM concentration [93]. This latter point might justify higher IC_50_ in *in vitro* studies, supporting time-dependent mechanisms that significantly differ between *in vitro* and *in vivo* experimental conditions. This hypothesis was indeed supported by our demonstration that prolonged metformin treatment of CMC xenografts resulted in reduced tumor size and growth arrest and depletion of CSC content, for much lower blood concentrations (about 40 μM) than required *in vitro* (about 10mM). The plasma levels obtained treating mice with metformin dissolved in drinking water to attain the dosage of 360 mg/kg body weight/day, which is comparable to therapeutic doses used for T2D as translated to humans (human equivalent dose [103]: corresponding to ~1,750 mg/day in an average-sized person of 60 kg), are therefore lower than the maximum recommended dose in humans (http://www.fda.gov/ohrms/dockets/dailys/02/May02/053102/800471e6.pdf).

Thus, *in vivo* dosing that induces a highly significant reduction of tumor growth is within human therapeutic range, even if the high safety profile and negligible side-effects of metformin could allow experimental doses over pharmacological concentrations. In very recent phase II and III clinical trials, non-diabetic women with BC received metformin at high dose (2 g/day) as adjuvant therapy [43,92], demonstrating that higher blood concentrations can be safely achieved.

Notably, our study demonstrates that the clonogenic potential of cells surviving long-term *in vivo* exposure to metformin, was markedly reduced as compared to untreated controls, indirectly confirming that metformin preferentially kills CSCs. Moreover, desensitization induced by prolonged exposure to metformin does not occur in CMC CSCs after prolonged *in vivo* treatment, since metformin still exhibits a strong and consistent antiproliferative action *in vitro* on cells derived *ex vivo* from both treated and untreated mice tumors. Above observations support metformin as an attractive agent for chemoprevention and use of low-dose for long period in combination with cytotoxic agents like DOX to kill both CSCs and the bulk of differentiated cancer cells.

## Conclusions

CSC-like subpopulation can be isolated from spontaneous canine mammary carcinomas, strongly highlighting the relevance of the comparative oncology model. Our data support CMC stem-like cells as a powerful model to provide information closer-to-primary cellular models for the identification of novel CSC targeting agents and the definition of biological behavior of stem-like cells in human BC. The demonstration of significant antitumor efficacy of metformin acting on CSC proliferative potential, along with the well-known safety profile support the ongoing evaluation of metformin in the clinical neo-adjuvant setting, although additional studies evaluating metformin effects in conjunction with standard treatments are needed to further focus the potential clinical benefits of this drug in BC. Companion animals could be investigated for possible translation to veterinary and human medicine, which may strengthen the use of naturally occurring CMC in dogs in comparative oncology trials.

## Additional file

Addtional file 1: Figure S1. ER-α expression and localization in canine mammary carcinomas. Immunohistochemistry staining in 5 representative cases of CMC tissues derived from 2 TPC (tubulopapillary carcinoma), 2 CC (complex carcinoma), and 1 CA (anaplastic carcinoma) showing different immunopositivity and localization for ER-α: case #1 and #2 = predominant cytoplasmic staining; case #3 = high nuclear immunoreactivity; case #4 = rare nuclear positivity; case #5 = negative staining.
