# Supplementary material for: In vitro and in vivo antiproliferative activity of metformin on stem-like cells isolated from spontaneous canine mammary carcinomas: translational implications for human tumors
Source: BMC Cancer. 2015 Apr 7;15:228. doi: 10.1186/s12885-015-1235-8 (PMC4397725; doi:10.1186/s12885-015-1235-8)
Supplement: Addtional file 1: Figure S1. — ER-α expression and localization in canine mammary carcinomas. Immunohistochemistry staining in 5 representative cases of CMC tissues derived from 2 TPC (tubulopapillary carcinoma), 2 CC (complex carcinoma), and 1 CA (anaplastic carcinoma) showing different immunopositivity and localization for ER-α: case #1 and #2 = predominant cytoplasmic staining; case #3 = high nuclear immunoreactivity; case #4 = rare nuclear positivity; case #5 = negative staining. [file 12885_2015_1235_MOESM1_ESM.pptx]

## Slide 1
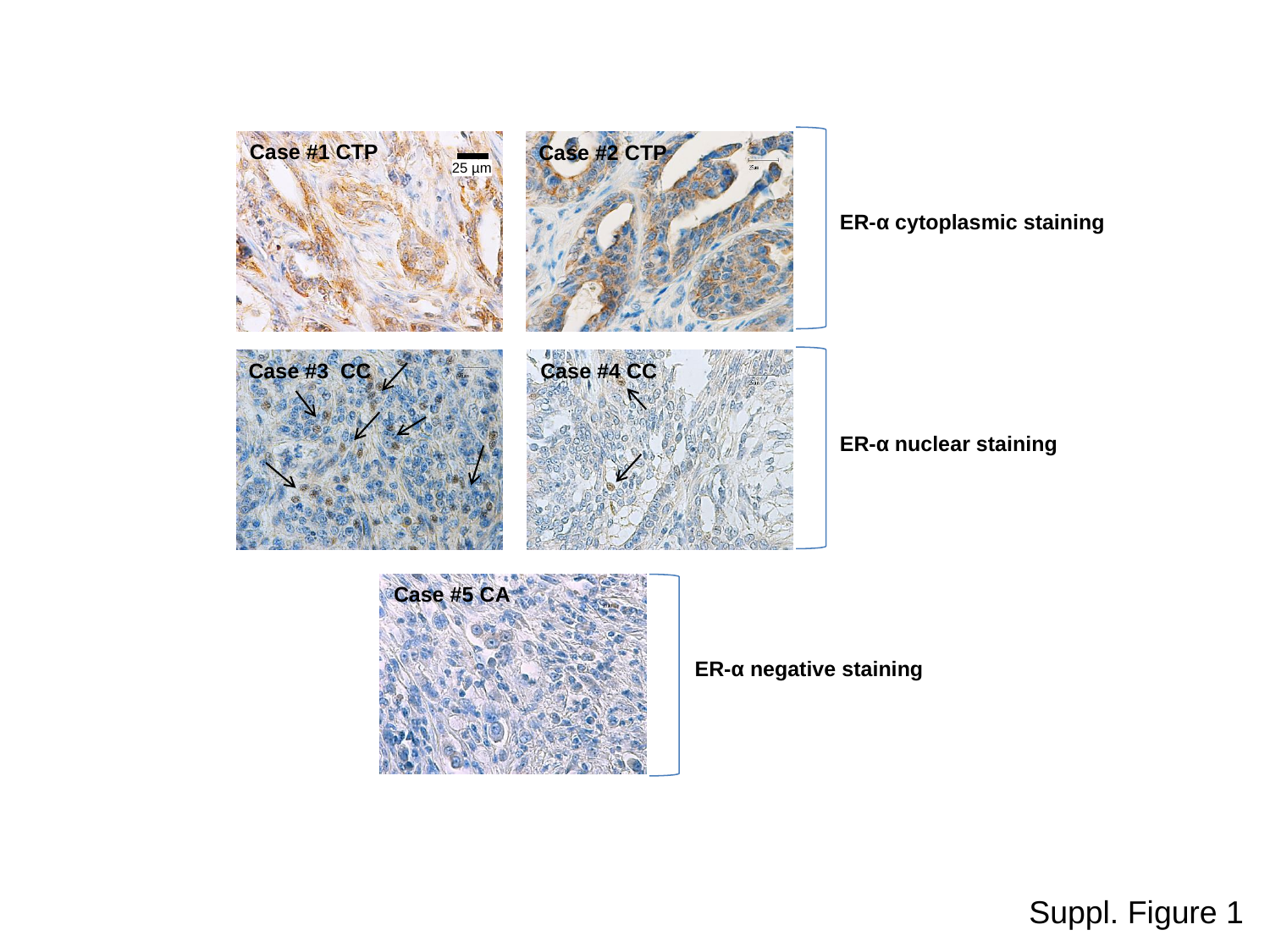

Case #1 CTP
Case #2 CTP
25 µm
ER-α cytoplasmic staining
Case #4 CC
Case #3 CC
ER-α nuclear staining
Case #5 CA
ER-α negative staining
Suppl. Figure 1
